# Exosomal-miRNas expression and growth factors released by mononuclear cells of CLAD patients in response to extracorporeal photopheresis

**DOI:** 10.1186/s12967-024-05045-6

**Published:** 2024-03-14

**Authors:** Sara Bozzini, Eleonora Bozza, Cecilia Bagnera, Claudia Del Fante, Eugenio Barone, Simona De Vitis, Mara De Amici, Giorgia Testa, Stefania Croce, Chiara Valsecchi, Maria A Avanzini, Rosalia Cacciatore, Cristina Mortellaro, Gianluca Viarengo, Cesare Perotti, Federica Meloni

**Affiliations:** 1 Department of Anesthesia and Intensive Care, Fondazione IRCCS Policlinico San Matteo, Pavia, Italy; 2 Department of Paediatric Oncoaematology/Cell Factory, Fondazione IRCCS Policlinico San Matteo, Pavia, Italy; 3 Immunohaematology and Transfusion Service, Cell Manipulation Laboratory, Fondazione IRCCS Policlinico San Matteo, Pavia, Italy; 4 Immuno-Allergology Laboratory of the Clinical Chemistry Unit and Pediatric Clinic, Fondazione IRCCS Policlinico San Matteo, Pavia, Italy; 5 Pediatric Clinic, Fondazione IRCCS Policlinico San Matteo, Pavia, Italy; 6 Immunohaematology and Transfusion Service, Fondazione IRCCS Policlinico San Matteo, Pavia, Italy; 7 Department of Cardio-Thoracic, Vascular Sciences and Public Health, University of Padua, Padua, Italy

**Keywords:** Exosomal-miRNas, Extracorporeal photopheresis, ECP, CLAD, T regulatory cells, Lung transplantation

## Abstract

**Background:**

CLAD (Chronic Lung Allograft Dysfunction) remains a serious complication following lung transplantation. Some evidence shows that portions of Extracorporeal Photopheresis (ECP)-treated patients improve/stabilize their graft function. In spite of that, data concerning molecular mechanisms are still lacking. Aims of our study were to assess whether ECP effects are mediated by Mononuclear Cells (MNCs) modulation in term of microRNAs (miRNAs) expression and growth factors release.

**Methods:**

Cells from leukapheresis of 16 CLAD patients, at time 0 and 6-months (10 cycles), were cultured for 48h ± PHA (10 ug/ml) or LPS (2 ug/ml). Expression levels of miR-146a-5p, miR-155-5p, miR-31-5p, miR181a-5p, miR-142-3p, miR-16-5p and miR-23b-5p in MNCs-exosomes were evaluated by qRT-PCR, while ELISA assessed different growth factors levels on culture supernatants.

**Results:**

Our result showed miR-142-3p down-regulation (p = 0.02) in MNCs of ECP-patients after the 10 cycles and after LPS stimulation (p = 0.005). We also find miR-146a-5p up-regulation in cells after the 10 cycles stimulated with LPS (p = 0.03). Connective tissue growth factor (CTGF) levels significantly decreased in MNCs supernatant (p = 0.04). The effect of ECP is translated into frequency changes of Dendritic Cell (DC) subpopulations and a slight increase in T regulatory cells (Treg) number and a significant decrease in CTGF release.

**Conclusions:**

ECP might affect regulatory T cell functions, since both miR-142 and miR-146a have been shown to be involved in the regulation of suppressor regulatory T cell functions and DCs. On the other side ECP, possibly by regulating macrophage activation, is able to significantly down modulate CTGF release.

**Supplementary Information:**

The online version contains supplementary material available at 10.1186/s12967-024-05045-6.

## Background

The long-term outcome of lung transplantation is significantly affected by the occurrence of chronic lung allograft dysfunction (CLAD). CLAD and its two main phenotypes, bronchiolitis obliterans syndrome (BOS) and restrictive allograft syndrome (RAS), have been redefined by the International Society of Heart and Lung Transplantation (ISHLT) [1]. Extracorporeal photopheresis (ECP), an immunomodulatory treatment based on the reinfusion of autologous UV-A irradiated peripheral blood leucocytes after the addition of 8-methoxypsoralen, is considered one of the most promising therapies for CLAD [2]. In particular, in BOS patients, ECP seems able to stabilize graft function without major side effects, even in highly compromised patients [3–5]. ECP has been shown to activate leucocytes and to induce some cell modifications, including apoptosis of T and NK cells [6–8]. It has been postulated that apoptotic T cells may be engulfed by activated monocytes that then differentiate into immature dendritic cells (iDC). After phagocytosis, iDC can maintain their immature phenotype or differentiate into mature plasmacytoid DC (pDC) through increased production of pro/anti-inflammatory cytokines. The latter leads to the presentation of antigens in a context of low co-stimulatory molecules with expansion of regulatory cell numbers and/or functions, increasing graft tolerance [9–11]. However, despite ECP treatment is being largely adopted in CLAD, some major questions are still open such as: (i) the exact mechanisms by which ECP exerts its therapeutic effect in CLAD patients, (ii) the possibility of predicting which patients will respond to ECP, (iii) the optimal treatment schedule and the impact of this treatment on patients’ quality of life. Few studies have investigated the ECP mechanism of action in CLAD, suggesting the occurrence of tolerogenic mechanisms but results are, so far, inconclusive [9–11]. The prediction of “ECP response” has also been attempted by clinical CLAD sub-phenotyping and, more recently, on a small cohort of ECP treated patients by the identification of some serum miRNAs, which appear to be dysregulated at ECP initiation and significantly affected by ECP only among treatment responders [12]. We observed a significant downregulation of circulating hsa-miR-155-5p, hsa-miR-146a-5p and hsa-miR-31-5p in BOS patients at the start of ECP when compared to healthy subjects. In responders, increased miR-155-5p and decreased miR-23b-3p expression levels at 6 months were found [12].

## Methods

### Aim, design and setting of the study

The aim of the present study was to assess whether ECP effects on miRNAs and cytokine dysregulation are due to the modulation of Mononuclear Cell (MNCs) activation and function.

This is a prospective pilot in vitro study, regarding Lung transplanted (LTx) patients who developed BOS and underwent ECP courses between 2018 and 2022. After BOS diagnosis (12% BOS stage 3, 19% BOS stage 2, 69% BOS stage 1), formulated according to recent ISHLT consensus documents [13], patients were treated by off-line ECP. All patients were treated with calcineurin inhibitors (Tacrolimus or Cyclosporin A) in combination with low-dose steroids; in addition, most patients also received Mycofeolate Mofetil. All patients had experienced a 3 month-course of low-dose Azithromycin before starting ECP.

16 adult patients with BOS were treated with ECP using the off-line method at the Apheresis Unit of the Immunohaematology and Transfusion Service of our hospital. Exclusion criteria were multi-organ transplantation and age ≤ 18. Successful treatment of BOS is usually defined as stabilization or slowing of FEV1 decline. In our study, non-responders were defined as patients who had a decline > 10% after ECP treatment. Conversely, responder were defined as patients with > 10% improvement of FEV1, compared with the value at the time ECP treatment was started, or stable patients with ≤ 10% improvement or ≤ 10% worsening of FEV1 compared to the value at the time of initiation of ECP treatment.

Plasma samples and MNCs were collected from all enrolled patients at two time-points: at ECP enrolment and after 10 cycles of treatments. For 5 responder patients it is possible to cryopreserve MNCs collected from leukapheresis (pre-irradiation) for further T regulatory cells (Tregs) and dendritic cells (DCs) evaluated by flow cytometry.

### ECP procedures

PBMCs were collected from the patient using a cell separator device, processing 1 blood volume. A complete cell count on the leukapheresis product was performed at the end of each collection, as per routine (quality control). Then, cells were irradiated (UV-A at 2 J/cmq; Macogenic, Macopharma, France) after the dilution with a saline solution and the addition of 8-methoxypsoralen (at 200 ng/mL concentration). Finally, the photoactivated PBMCs were immediately reinfused into the patient [14]. During the entire ECP procedure, vital parameters, such as blood pressure, heart rate and oxygen saturation, were monitored basing on the patient’s clinical status. Any adverse event related to the procedure was registered [6, 14].

Each ECP cycle comprises two treatment that are performed every on consecutive days. ECP treatment schedule consisted in 2 procedures weekly for 2 weeks followed by cycles every other week and then 1 cycle a month. The schedule of ECP was reported in Additional file 1: Figure S2.

### Mononuclear cell culture

MNCs, obtained from leukapheresis at the first and the tenth ECP cycle, were cultured for 48 h in RPMI supplemented with 10% (v/v) heat-inactivated fetal bovine serum (FBS) and 10 ug/ml of Phytohaemagglutinin (PHA) or RPMI medium with 10% FCS and 2 ug/ml of Lipopolysaccharides (LPS). After incubation, the supernatant was collected and stored at – 20 ℃ until analysis.

In a subpopulation of responder patients (N = 5), leukapheresis cells was used for the determination of DCs and 20 × 10^6^ cells were subjected to cryopreservation for subsequent determination of Treg cells numbers.

### miRNAs selection

For the present study, we selected four miRNAs previously identified as being associated with BOS and/or ECP response in serum [12] and miRNAs previously reported in proliferation and function regulation of the main immune cell types of the innate and adaptive immunity [15, 16]. In particular, we investigated the relative expression of hsa-miR-155-5p, hsa-miR-146a-5p, hsa-miR-31-5p, hsa-miR-181a-3p, hsa-miR-16-5p, hsa-miR-142-3p and hsa-miR-23b-5p, reported to be involved in the regulation of DCs, B ad T cell immunosoppressive properties. These cells are known to play a critical role in maintaining tolerance and/or driving graft rejection.

### miRNAs analysis

Exosome-derived RNA were obtained using the exoRNeasy Serum/Plasma Midi Kit (Qiagen) from 1 mL MNCS culture supernatant. The kit provides exosome isolation followed by a total RNA isolation procedure.

Complementary DNA (cDNA) was synthesized with miRCURY LNA RT Kit (Qiagen) at 42 ℃ for 60 min and 95 ℃ for 5 min. For the quality control of differences in RNA extraction or RT efficiencies, a synthetic cel-miR-39 was utilized as spike-in control RNA. Real-time PCR analysis was performed to evaluate miRNAs’ expression levels using miRCURY LNA miRNA PCR-specific Detection Probe and miRCURY LNA SYBR Green PCR Kit (Qiagen) with a LightCycler 480 (Roche, Switzerland), according to the manufacturer’s recommendations. Thermal cycling conditions consisted of initial denaturation at 95 ℃ for 10 min, followed by 45 cycles of 95 ℃ for 10 s followed by 60 ℃ for 1 min. The threshold cycle (Ct) was defined as the fraction cycle number at which fluorescence exceeded the given threshold. The stable Ct values of cel-miR-39 obtained from all spike-ins indicated successful RNA isolation, reverse transcription and qPCR detection system. Each experimental condition was performed in triplicate. Relative quantifications were calculated with the comparative Ct method.

### Normalization analysis and identification of endogenous reference

We first screened for the most stability expressed miRNAs. miRNAs were processed by NormFinder (http://moma.dk/NormFinder-software), which works with an algorithm for linear data to determine the most stable normalization candidate gene [17]. The tool calculates a stability value (SV) for every single candidate. The data output come with the stability values for all candidates together with standard deviations (SD) and announces the most stable candidate gene. The lower stability value identified the more stable and it is the expression of the corresponding candidate gene. Splitting data into study groups, results in additional intra- and intergroup variation and determination of the best endogenous normalizer combination.

### Growth factor arrays

Connective tissue growth factor (CTGF), transforming growth factor-beta (TGF-β), Vascular Endothelial Growth Factor (VEGF), Fibroblast growth factors (FGF) and Platelet-Derived Growth Factor (PDGF) levels were determined on culture supernatant by ELISA (Abcam) following the manufacturer’s instructions and the results were expressed as pg mL − 1.

### T regulatory cells (tregs) and dendritic cells (DCs)

Tregs were evaluated by flow cytometry on cryopreserved MNCs collected from leukapheresis (pre-irradiation). Cells were thawed and suspended in RPMI supplemented with 10% heat-inactivated fetal calf serum (Euroclone, Milan, Italy). For cell-surface staining, anti CD4, CD127 and CD25 antibodies (Becton Dickinson, Milan, Italy) were used. After incubation at + 4 ℃ for 30 min, cells were than treated with fixation/permeabilization buffer (eBioscience, Waltham, MA, USA) at + 4 ℃ for 40 min. After three washings, intracellular staining with forkhead box P3 transcription factor (FoxP3) specific antibody (eBioscience, ThermoFisher Scientific, Waltham, MA, USA) was carried out at + 4 ℃ for 30 min. Appropriate isotype-matched controls were used. Acquisition and analysis were performed by Canto II flow Cytometer (Becton Dickinson). Tregs were defined as CD4 + CD127- CD25 + cells expressing FoxP3, and expressed as percentage.

For DCs, a sample of 100 µl obtained from leukapheresis (pre-irradiation) was diluited 1:10 in PBS Buffer (BeckmanCoulter Inc., USA), then 100 µl of solution was incubated with monoclonal antibodies (mAbs) for 15 min in the dark at room temperature. The staining mAbs included: 20 µl Lineage cocktail (CD3 CD14 CD16 CD19 CD20 CD56)-FITC, 20 µl HLA DR-PE, 5 µl CD45-V500, 5 µl CD11c-APC, 2 µl CD123-BV605 (BD Biosciences, San Jose, CA, USA) according to the manufacturer’s instructions. After 10 min of incubation in presence of 2 ml of Ammonium Cloride/Lysing buffer 1 × the sample, completely clear, was analyzed. The acquisition of events was performed using a medium flow rate, with 100000 CD45 + events as stop criteria. The analysis was performed as follows: cells were gated on CD45; after exclusion of Lineage Cocktail positive cells, HLA DR + population was selected and finally divided into plasmacytoid DCs (CD123 +), conventional DCs (CD11c +) and immature DCs (CD123-/CD11c-).

### Plasma IL-10 determination

The plasma Interleukin-10 (IL-10) was titled using a commercial kit (Human Immunoassay, R&D Systems, Minneapolis, MN), with a quantitative enzyme immunoassay technique, according to the Manufacturer’s Instructions. The results are expressed in pg mL − 1.

### Statistical analysis

The mean and standard deviation or median and interquartile range were presented for continuous variables, and numbers and percentages were presented for categorical variables. For continuous variables, groups were compared using parametric or non-parametric tests, according to data distribution. Correlations were calculated using Spearman’s correlation test. One-way analysis of variance was used to calculate the differences in candidate reference genes between the patients and controls. Statistical analyses were performed using GraphPad Prism (GraphPad Software, Inc., San Diego, CA, USA). All statistical tests were two-sided, and a p-value < 0.05 was considered statistically significant.

## Results

### Demographic and clinical features of patients

Sixteen patients were included in the analysis with a mean age at lung transplantation of 44 ± 16 years. 69% of patients were males (69/31 M/F). The most represented underlying diagnosis was idiopathic pulmonary fibrosis (IPF, 25%), followed by cystic fibrosis (CF, 12.5%) and lung fibrosis secondary to asbestos/alveolitis (12.5%). In Table 1 were summarized features of patients classified in responder and non-responder.

**Table 1:** demographic and clinical features of patients included in the study

	BOS patients (n = 16)	Responder patients (n = 11)	Non responder patients (n = 5)
Males (n, %)	11 (69%)	8 (73%)	3 (60%)
Age at transplantation (mean ± DS)	44 ± 16	45 ± 17	41 ± 9
Underlying diagnosis (n, %):			
- IPF	4 (25%)	2 (18%)	2 (40%)
- Cystic Fibrosis	2 (12.5%)	1 (9%)	1 (20%)
- Lung Fibrosis secondary to asbestos/alveolitis	2 (12.5%)	1 (9%)	1 (20%)
- Other	8 (50%)	7 (64%)	1 (20%)
Bilateral lung transplantation (n, %)	12 (75%)	7 (64%)	5 (100%)

According to the stabilization or improvement of lung function from basal ECP entry value, 5 (31%) patients were classified as non-responders and 11 (69%) patients were deemed as responders after 6 months of ECP treatment.

### Candidate miRNAs for endogenous normalization

We investigated the relative expression of hsa-miR-155-5p, hsa-miR-146a-5p, hsa-miR-31-5p, hsa-miR-181a-3p, hsa-miR-16-5p, hsa-miR-142-3p and hsa-miR-23b-5p, involved in maintaining tolerance and/or driving rejection of grafts as knowing regulator of immunosoppressive properties of DCs, B cells ad T cells [15, 16].

To identify potential miRNA references for exosomal RNA quantification, we screened for the most stable expressed miRNAs by NormFinder. Expression levels of the small nuclear RNA RNU6 were also included in the normalization analysis.

Based on NormFinder analysis, miR-16-5p ranked as the most stable miRNA and then it was used for normalization (Additional file 1: Figure S1).

### Expression levels of selected miRNAs in unstimulated/stimulated MNCs-derived exosomes

Following our previous results on the levels of circulating miRNAs [12], in order to define if ECP effects are mediated by modulation of MNCs in term of differential miRNA expression and release. The present study represents an extension of the previous one [12] and it regard patients subjected to ECP using the off-line method at the Apheresis Unit of the Immunohaematology and Transfusion Service of our hospital.

In the present study, we determined the expression levels of miR-155-5p, miR-146a-5p, miR-31-5p, miR-181a-3p, miR-142-3p and miR-23b-5p in unstimulated/stimulated MNC-derived exosomes. In Additional file 1: table S1, we reported the overall results of the seven miRNAs in the studied population.

**Fig. 1:**
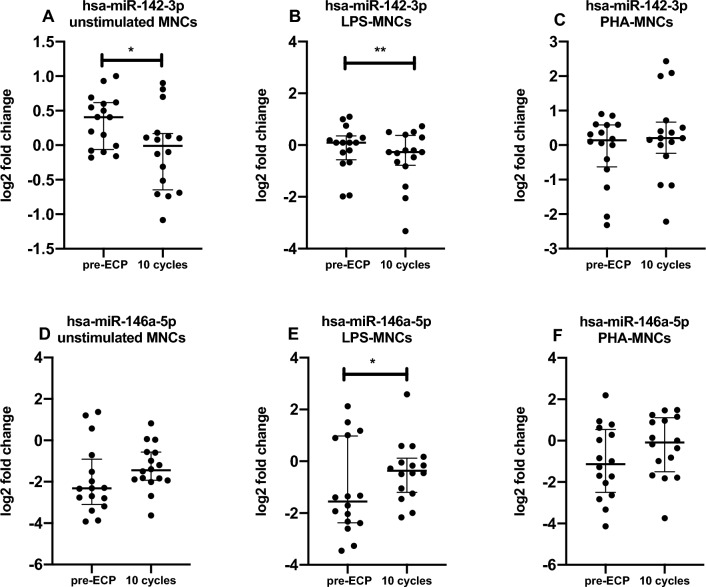
Quantitative expression of (**A**, **B**, **C**) miR-142-3p and (**D**, **E**, **F**) miR-146a-5p in exosome of MNCs patients before ECP therapy and after 10 cycles of treatment. Relative expressions were expressed as log2 transformed values. *p < 0.05, ** p < 0.01

After 10 cycles of ECP we observed that the release in exosomes of miR-142-3p by unstimulated MNCs was down-regulated compared to pre-treatment levels (Fig. 1A,  p =0.0215). This was also replicated when MNCs were stimulated with LPS (Fig. 1B, p = 0.0048), but not with PHA (Fig. 1C).

On the other hand, miR-146a-5p resulted up-regulated by ECP when MNCs were stimulated by LPS (Fig. 1E, p = 0.0329). No significant difference was detected in miR-146a-5p levels when MNCs were unstimulated or stimulated with PHA (Fig. 1D–F).

**Fig. 2:**
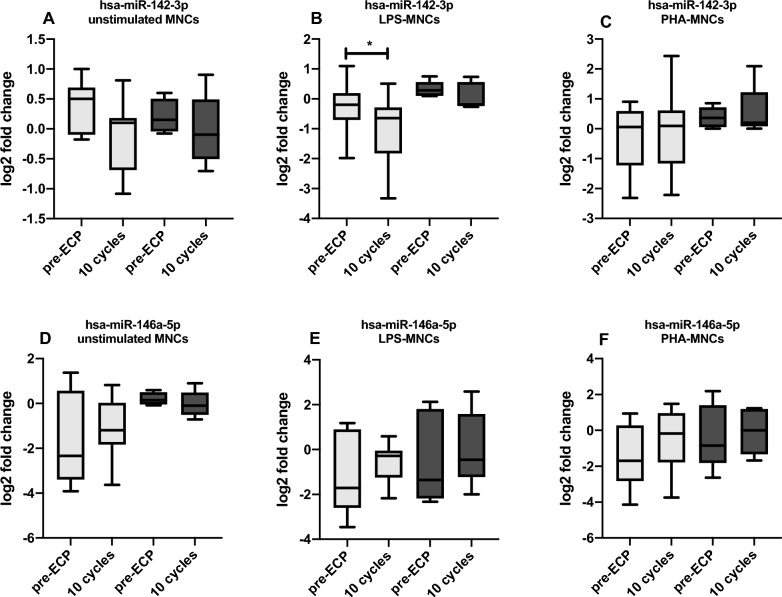
Quantitative expression of (**A**, **B**, **C**) miR-142-3p and (**D**, **E**, **F**) miR-146a-5p assessed by qRT-PCR in responder (light grey box) and non responder (grey box) patients. Relative expressions were expressed as log2 transformed values. *p < 0.05

In order to investigate whether any miRNAs could predict the response to ECP, we compared their baseline levels in MNCs exosomes of patients who showed a clinical response/no response to therapy at last follow up (Fig. 2). However, we could not detect any significant difference between these two groups of patients. Limiting the analysis to responders, a significant downregulation of hsa-miR-142-3p in exosome was present when MNCs were stimulated with LPS (p = 0.0117).

### Growth factor levels in supernatant of unstimulated/stimulated MNCs

**Fig. 3:**
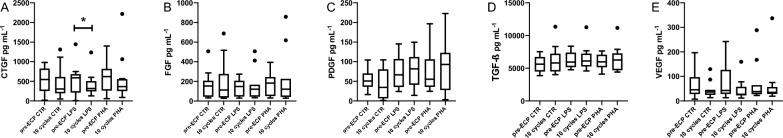
Growth factor levels in supernatant of unstimulated/stimulated MNCs. *p < 0.05

Growth factor levels were assessed in supernatant of unstimulated/stimulated MNCs at the two distinct time points: pre-ECP and after 10 cycles (Fig. 3). CTGF release by LPS-stimulated MNCs was significantly reduced by ECP (p = 0.0475, Fig. 3A).

### Dendritic cell subsets and regulatory T cells

**Fig. 4:**
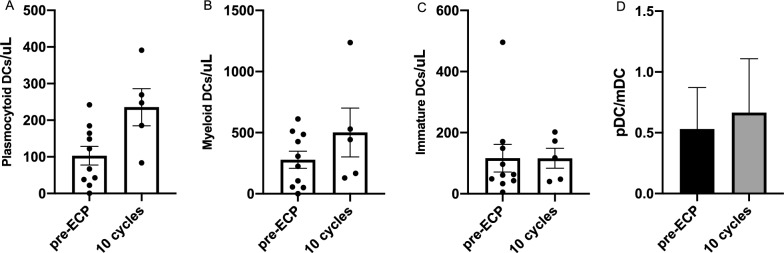
DCs subpopulations at baseline and after ECP: CD123 + plasmacytoid DC (**A**), CD11c + myeloid DC (**B**) and immature DCs (**C**). Plasmacytoid and myeloid DC ratio before and after 10 ECP cycles (**D**)

We next sought to understand mechanism by which ECP and miRNAs mitigates CLAD in a subpopulation of responder patients (N = 5). Our data demonstrated how the effect of ECP was translated into frequency changes of DC subpopulations by monitoring the rate of CD123 + plasmacytoid DC (pDC) (Fig. 4A) and CD11c + myeloid DC (mDC) (Fig. 4B). We observed that both pDC and mDC were increased in responder patients over 10 cycles of ECP treatment, which however does not reach significant differences, but the pDC/mDC ratio remained unaltered (Fig. 4D). Our data also show how the number of immature DCs remains unchanged before and after treatment (Fig. 4C), underlining the association of DC maturation to the therapeutic mechanism of ECP.

**Fig. 5:**
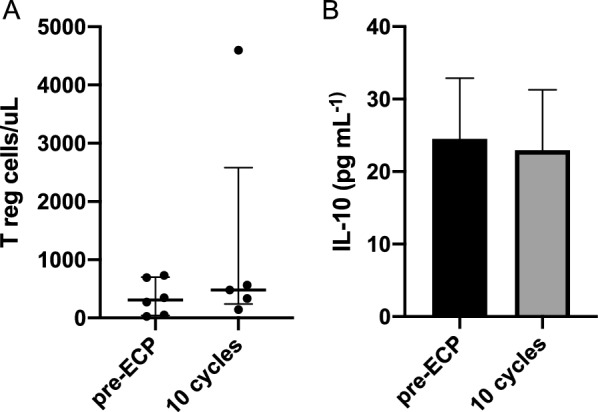
Treg frequency (**A**) and plasma IL-10 (**B**) in responder patients, pre-ECP and after 10 cycles

ECP treatment does not induced an increase in Treg numbers (Fig. 5A) as well as in IL-10 plasma levels (Fig. 5B) after 10 cycles.

## Discussion

Epigenetic changes, including miRNAs deregulation, have been suggested to play a significant role in development of CLAD in LTx recipients. Many studies have tried to identify miRNA ideal candidate, and the downstream pathways implicated in the bronchiolar fibro-obliterative process [17]. Different studies evaluated the immunomodulatory effect of ECP therapy in patients after solid organ transplant rejection [18–22], but few studies have explored the molecular regulation associated with ECP treatment [12].

Our data on patients with obstructive CLAD phenotype show that ECP is able to up-regulate the release of miR-146a-5p by MNCs upon stimulation with LPS. These data are consistent with those previously observed in the serum of ECP-treated patients where we found a different expression profile of specific circulating immunoregulatory miRNAs. In fact, in patients with BOS at the time of ECP enrolment miR-146a-5p was significantly downregulated in serum compared to healthy controls [12]. Within the BOS patient population, we found a non-significant trend towards an increase, was observed for post-ECP miR-146a serum levels. Based on the present results, we hypothesize that miR-146a-5p levels in serum were potentially due to its release by MNCs and achieved significant differences when were directly assessed on the exosomes released by MNCs, as in the present study. The role of miR-146a has been documented in the negative regulation of immune responses, particularly of myeloid cells due to limiting TNF receptor-associated factor 6 (TRAF6) stimulation and interleukin receptor-associated kinase 1/2 (IRAK1/ 2)-mediated signaling in inflammatory conditions [23, 24]. MiR-146a expression has been reported to be elevated in Tregs and was induced upon activation [25]. Increased expression of miR-146a has also been observed in human monocytes in response to TLR4 stimulation by lipopolysaccharides through direct NF-κB–mediated induction [24]. In addition, miR-146a is crucially involved in the survival and TLR-induced maturation of pDCs, which influence their ability to induce CD4 + T cells proliferation and IFN-γ production [26].

In the present study, we also observed a significant upregulation of miR-142-3p by unstimulated MNCs and after culturing in the presence of LPS. Several published studies demonstrated that miR-142-3p plays a role in the modulation of Treg function and in particular, its down-regulation confers suppressor functions to Treg cells [27]. In mice, miR-142 is constitutively expressed in immature bone marrow DCs, and following LPS activation, its expression is decreased [28]. An association between miR-142 and monocyte-derived DCs (moDCs) of patients with Systemic Lupus Erythematosus (SLE) has been described in the literature [29]. In the same study, the overexpression of miR-142-3p caused the elevation of IL-6 and TNF-α, leading to a decrease of CD4 + CD25 + Foxp3 + Tregs (which have anti-inflammatory effect), and an imbalance of IL-17 and IL-10. Therefore, overexpression of miR-142-3p in moDCs suppressed Tregs increase, which correlated with a reduced capability to suppress responder T cell proliferation and might thereby contribute to the development of SLE [29]. This therefore induces us to speculate that a decrease in release of miR-142-3p by MNCS in ECP treatment might contribute to modulating the activity or the frequency of Tregs clones.

Previous studies indicate a key role played by DCs and Tregs in the immunomodulatory mechanism of ECP and suggest that ECP induces Tregs expansion and/or tolerogenic DCs [30–32]. Moreover, it was demonstrated that ECP might influence the frequency of circulating Tregs [31]. In the present study, we observed a slight increase in Tregs in responder patients, even in the absence of an increase in plasma IL-10 levels. Our data show how the effect of ECP is translated into frequency changes in DC subpopulations with an increase of both pDC and mDC. Although the lack of statistical significance indicates the need for analysis in a larger cohort to ascertain the validity of these findings, higher pDC and mDC support an ECP-induced effect.

These miRNAs may be involved in the modulation of other regulatory cell clones. Indeed, published data indicate that miRNAs are also involved in modulating transcriptional factors to become complex regulatory networks that regulate the Myeloid/Derived Suppressor Cells (MDSC) [33]. In melanoma, miR-146a was responsible for the conversion of monocytes into MDSC (CD14 + HLA-DR neg cells) mediated by melanoma extracellular vesicles and were shown to recreate MDSC features upon transfection [34], suggesting that its levels can influence the fine-tuning of pro- or anti-inflammatory pathways, depending on the cell type. Interesting future developments could be aimed at the search for myeloid suppressor cells in patients who will undergo ECP together with their correlation with flow cytometric markers of suppressive activity.

Lastly, we also detected a significant variation in CTGF release, which resulted significantly down regulated by ECP treatment, when cells were stimulated by LPS. CTGF is an important mediator in several fibrotic disorders. CTGF in plasma and urine has previously been proposed as a biomarker monitoring tool to measure the extent of ongoing fibrosis in several fibrotic disorders and was correlated with disease severity [35]. This growth factor plays a relevant role in post-transplant fibrogenesis and its expression in Broncho Alverolar Lavage (BAL) has been recently shown to be associated with fibrosis in CLAD, although no significant difference in plasma CTGF levels was found [36]. This data, if confirmed in a larger cohort, could suggest a possible interference of ECP in graft fibrogenesis via modulation of CTGF release.

## Conclusion

We can infer that ECP might affect regulatory T cell functions, since both miR-142 and miR-146a have been shown to be involved in the regulation of suppressor regulatory T cell functions and DCs. On the other side ECP, possibly by regulating macrophage activation, is able to significantly down modulate CTGF release. This preliminary study opens some future directions, including a better definition of miRNAs role in DC subpopulations and Treg functions and the confirmation of their implication in the ECP mechanism of action.

### Supplementary Information

**Additional file 1: Figure S1.** Candidate miRNAs for endogenous normalization. Based on NormFinder analysis, miR-16-5p ranked as the most table miRNA. **Figure S2.** Schematic representation of ECP schedule: ECP treatment schedule consisted in 2 procedures weekly for 2 weeks followed by cycles every other week and then 1 cycle a month. Each ECP cycle comprises two treatment that were performed every on consecutive days. **Table S1.** overall results of the seven miRNAs in the studied population. Relative expressions were expressed as log2 transformed values.

## Data Availability

All data generated or analysed during this study are included in this published article and its Additional files.
