# Supplementary material for: Exosomal-miRNas expression and growth factors released by mononuclear cells of CLAD patients in response to extracorporeal photopheresis
Source: J Transl Med. 2024 Mar 14;22:276. doi: 10.1186/s12967-024-05045-6 (PMC10938790; doi:10.1186/s12967-024-05045-6)
Supplement: Supplementary file 1 — Additional file 1: Figure S1. Candidate miRNAs for endogenous normalization. Based on NormFinder analysis, miR-16-5p ranked as the most table miRNA. Figure S2. Schematic representation of ECP schedule: ECP treatment schedule consisted in 2 procedures weekly for 2 weeks followed by cycles every other week and then 1 cycle a month. Each ECP cycle comprises two treatment that were performed every on consecutive days. Table S1. overall results of the seven miRNAs in the studied population. Relative expressions were expressed as log2 transformed values. [file 12967_2024_5045_MOESM1_ESM.docx]

**Figure S1:** Candidate miRNAs for endogenous normalization. Based on NormFinder analysis, miR-16-5p ranked as the most table miRNA.

**Figure S2:** Schematic representation of ECP schedule: ECP treatment schedule consisted in 2 procedures weekly for 2 weeks followed by cycles every other week and then 1 cycle a month. Each ECP cycle comprises two treatment that were performed every on consecutive days.

|  |  | Baseline | | | | 10 cycles | | | | p-value |
| --- | --- | --- | --- | --- | --- | --- | --- | --- | --- | --- |
|  |  | 25% Percentile | Median | 75% Percentile | 25% Percentile | | Median | 75% Percentile |  | |
| CTR | hsa-miR-146a-5p | -3.093 | -2.323 | -0.9125 | -1.928 | | -1.448 | -0.57 | 0.1586 | |
|  | hsa-miR-155-5p | -6.06 | -5.71 | -5.125 | -5.42 | | -5.04 | -4.55 | 0.2359 | |
|  | hsa-miR-23b-5p | -5.042 | -3.63 | -2.56 | -5.74 | | -3.55 | -2.74 | 0.6256 | |
|  | hsa-miR-31-5p | -7.67 | -5.945 | -4.033 | -9.97 | | -6.572 | -4.74 | 0.7312 | |
|  | hsa-miR-181a3p | -13.15 | -10.48 | -7.11 | -11.04 | | -6.39 | -6 | 0.07 | |
|  | hsa-miR-142-3p | -0.0625 | 0.4075 | 0.615 | -0.6456 | | -0.01 | 0.1675 | **0.0215** | |
| PHA | hsa-miR-146a-5p | -2.493 | -1.142 | 0.535 | -1.508 | | -0.09 | 1.103 | 0.0867 | |
|  | hsa-miR-155-5p | -2.273 | -1.345 | -0.58 | -2.623 | | -1.045 | 0.0075 | 0.9371 | |
|  | hsa-miR-23b-5p | -5.69 | -4.081 | -3.263 | -5.964 | | -4.26 | -1.748 | 0.2812 | |
|  | hsa-miR-31-5p | -8.359 | -6.251 | -5.51 | -7.21 | | -6.453 | -4.59 | 0.8720 | |
|  | hsa-miR-181a3p | -11.3 | -9.7 | -8.365 | -8.883 | | -5.759 | -3.853 | 0.081 | |
|  | hsa-miR-142-3p | -0.6275 | 0.14 | 0.5875 | -0.24 | | 0.2 | 0.6667 | 0.1582 | |
| LPS | hsa-miR-146a-5p | -2.375 | -1.555 | 0.9764 | -1.2 | | -0.37 | 0.115 | **0.0329** | |
|  | hsa-miR-155-5p | -4.233 | -3.44 | -3.317 | -4.135 | | -3.633 | -3.458 | 0.524 | |
|  | hsa-miR-23b-5p | -5.117 | -3.41 | -1.403 | -5.519 | | -3.649 | -2.61 | 0.207 | |
|  | hsa-miR-31-5p | -6.608 | -5.396 | -3.505 | -7.116 | | -5.955 | -5.033 | 0.2753 | |
|  | hsa-miR-181a3p | -11.7 | -10.56 | -9.8 | -9.76 | | -7.26 | -5.745 | 0.0515 | |
|  | hsa-miR-142-3p | -0.565 | 0.0975 | 0.355 | -0.7775 | | -0.2689 | 0.375 | **0.0048** | |

**Table S1:** overall results of the seven miRNAs in the studied population. Relative expressions were expressed as log2 transformed values.
